# Alpha gal syndrome - A rare case report in South India

**DOI:** 10.6026/973206300210982

**Published:** 2025-05-31

**Authors:** Suganya Sribalaji, Asmathulla S., Manavalan J.

**Affiliations:** 1 Department of Biochemistry, Sri Manakula Vinayagar Medical College and Hospital, Kalitheerthalkuppam, Madagadipet, Puducherry, India; 2 Department of Biochemistry, AIIMS Madurai, Tamilnadu, India

**Keywords:** Tick bite, alpha gal allergy, alpha gal syndrome

## Abstract

A 31-year-old female presented with complaints of itching with previous history of 5-6 anaphylactic reactions since last one month. Her symptoms were preceded (3-6 hours) with intake of red meat. She suffered one tick bite (probably a lone star tick) one month before the allergic episodes. Laboratory tests showed elevated serum IgE levels. She was suspected to suffer from Alpha gal syndrome. It is a disease causally linked to bites from ticks. Although cases of alpha-gal allergy have already been reported in a few European countries, to our best knowledge, no cases have been reported so far in South India. She was managed with steroids and dietary restrictions.

## Background:

Alpha-gal syndrome is an immunoglobulin E (IgE)-dependent allergy to galactose-α-1, 3-galactose (alpha-gal), resulting in a delayed anaphylactic reaction to the consumption of red meat. The syndrome was first described in 2008 in the Southeastern United States and is causally linked to bites from ticks. There have been case reports in Australia, South Africa, Asia (Japan, South Korea) and in a few European countries like Sweden, Norway, France, Italy, Spain, Germany, Switzerland and Austria [1, 2, 3, 4, 5-6]. However, to our best knowledge, no cases have been reported so far in South India. If undiagnosed, the alpha-gal syndrome may result in severe hypersensitivity reactions to foods and drugs [7]. This syndrome can be easily overlooked due to relatively low awareness among healthcare professionals as well as some untypical clinical features. Therefore in this paper, we intend to describe a case of alpha-gal syndrome diagnosed in South India and highlight some important clinical implications to draw clinicians' attention to the importance of early diagnosis and management.

## Case presentation:

A 31-year-old female presented with complaints of itching all over the body. She also had previous history of 5-6 anaphylactic reactions in the last one month. The typical reaction involves pruritic urticarial rash on the forearm, lower limb and upper and lower lip. Three episodes of shortness of breath were also present. Symptoms occur after 3-6hrs after ingestion of the red meat (mutton). The patient treated with intravenous steroidal and antihistamines. She reported no symptoms after the consumption of chicken. She suffered one tick bite (probably a lone star tick) one month before the episodes of allergy. She is a known case of sarcoidosis but on good prognosis and not on any treatment at present. Laboratory tests revealed a significantly elevated level of IgE antibodies (more than 400 IU/L, reference range < 175 IU/L) which was determined by ELISA. She was instructed to avoid mammalian meat and other products containing alpha-gal. Follow up of the case revealed that she did not develop such further episodes due to her strict compliance to the dietary advice.

## Discussion:

Galactose-α-1, 3-galactose (alpha gal) is an oligosaccharide expressed on the cells of non-primate mammals. In the course of evolution, Homo sapiens have lost the ability to synthesize alpha-gal, thereby the carbohydrate become immunogenic to humans [7]. The symptoms are primarily seen after the consumption of meat from non-primate mammals, such as beef, pork and lamb. Less frequently, they appear following the intake of products containing gelatine while poultry and fish tend to be well tolerated [8]. The digestion of alpha-gal-rich glycoproteins and glycolipids, may take quite a long time. Because of this, the first symptoms of red meat allergy may develop even several hours after consumption (ranging from 3 to 7 hours).

## Mechanism:

Allergic reaction to α-Gal occurs after tick bites. Tick saliva contains proteins glycosylated with α-Gal and α-Gal containing glycolipids. α-Gal on proteins is recognized by memory B cells and process them to present the resultant peptides to naT cells. Glycolipids might be recognized by iNKT cells, which inturn produces IL-4. In the IL-4-rich tissue, T cells then induce the class switch recombination of B cells leading to the production of IgE to α-Gal, which then binds to basophils and mast cells. When sensitized individuals ingest red meat containing α-Gal bound to proteins and lipids, these glycolipids are incorporated into lipid micelles. Pancreatic lipase, an enzyme active at water-lipid interfaces, hydrolyzes the triglycerides inside the micelle into free fatty acids, mono- and diglycerides, which are absorbed by enterocytes. About 4 h later, processed lipids, packed in chylomicrons and presumably coated with α-Gal molecules, are released into the lymph via the lacteal vein. When chylomicrons reach the bloodstream and tissues, they encounter basophils and mast cells coated with IgE antibodies to α-Gal. The α-Gal moieties displayed on the surface of chylomicrons can then cause the cross-linking of IgEs and the subsequent degranulation of basophils and mast cells leading to a systemic allergic reaction [9]. Conditions like AGS are rare and can be missed as an initial diagnosis in many patients [10]. The syndrome is strikingly regional; reflecting the important role of tick bites in sensitization, and is more common in demographic groups at risk of tick exposure [11].

## Conclusion:

Alpha-gal syndrome is an allergic manifestation to the red meat proteins after a tick bite reported often in European countries. This case is presented as it is the first case reported from South India. This provides the healthcare system with information to suspect this disorder in the future. The early diagnosis will facilitate effective management of the symptoms and prevent potentially life-threatening complications.
